# A multicenter prospective comparative study evaluating cataract surgery and endoscopic cyclophotocoagulation either with or without iStent *inject* implantation in Brazilian patients with glaucoma

**DOI:** 10.1007/s10792-022-02563-4

**Published:** 2022-10-23

**Authors:** Francisco E. Lima, João C. Geraissate, Marcos P. Ávila

**Affiliations:** 1 Federal University of Goiás, Goiânia, Brazil; 2 Centro Brasileiro de Cirurgia de Olhos, Avenida T-2 número 401, Setor Bueno, Goiânia, 74210-010 Brazil; 3 Centro Brasileiro da Visão, Brasília, Brazil

**Keywords:** Trabecular bypass, MIGS, ECP, Combination, Phacoemulsification

## Abstract

**Purpose:**

Compare 12-month (12 M) safety and efficacy of endoscopic cyclophotocoagulation (ECP) + cataract surgery (Group 1) versus ECP + cataract surgery + iStent *inject* trabecular micro-bypass implantation (Group 2) in Brazilian patients with open-angle glaucoma (OAG).

**Methods:**

This prospective, multicenter, comparative case series included patients with OAG and cataract who were randomized to receive treatment in Group 1 or Group 2. Outcomes included mean and percent reduction versus preoperative in intraocular pressure (IOP) and number of glaucoma medications; visual acuity; occurrence of adverse events; and rate of secondary surgeries.

**Results:**

Preoperatively, Groups 1 and 2 had similar mean IOP (mean ± standard deviation 22.1 ± 3.6 and 22.0 ± 2.5 mmHg, respectively) and mean number of medications (3.3 ± 0.6 and 3.4 ± 0.6 medications, respectively). At all follow-up timepoints through 12 M, both groups achieved significant IOP and medication reductions versus preoperative (IOP *p* < 0.001 and number of medications *p* < 0.001 for both groups). At 12 M, IOP reductions were 24.2% (Group 1) and 43.6% (Group 2) (*p* < 0.001); mean medication reductions were 50.2% and 71.5%, respectively. Mean postoperative IOP and number of medications were higher in Group 1 than Group 2 (IOP *p* < 0.01 all visits, medication *p* < 0.01 at 6 M and 12 M). Adverse events were generally mild and infrequent in both groups.

**Conclusion:**

Both treatment groups (ECP + phacoemulsification, with/without iStent *inject* implantation) achieved significant and safe reductions in IOP and medications versus preoperative in Brazilian OAG patients. Percent reductions were significantly greater, and mean IOP and medications were significantly lower, in the group receiving iStent *inject*.

**Clinical trial registration (CTR):**

CAAE project identification #20053019.5.0000.5078. Protocol #3.587.147. Clinical Trial Database of the Federal University of Goiás, Brazil. Registration Date: September 19, 2019.

**Supplementary Information:**

The online version contains supplementary material available at 10.1007/s10792-022-02563-4.

## Introduction

Glaucoma is the leading cause of irreversible blindness worldwide [1]. A meta-analysis of 50 population-based studies encompassing over a quarter of a million individuals estimated the global prevalence of glaucoma for those aged 40 to 80 years at 3.5%. The number of people in this age group with glaucoma was estimated at 64.3 million in 2013 and is anticipated to increase to 111.8 million in 2040 [1].

Medical and surgical therapies for treating glaucoma focus on lowering intraocular pressure (IOP), currently the only known modifiable risk factor [2]. Although medications are moderately effective and safe, chronic treatment with topical ocular hypotensive agents can be associated with deleterious effects to the ocular surface, issues with patient adherence and persistence to their prescribed regimens, and reduced chance for success with subsequent surgical procedures [3–8].

In the surgical realm, phacoemulsification alone often is associated with modest IOP reduction due to an increase in outflow facility [9]; however, these effects appear to be modest and impermanent [10]. Traditional filtration surgeries (e.g., trabeculectomy and tube implants) can dramatically reduce IOP, but often are associated with considerable morbidity [11–13]. In the past decade, a new class of procedures, micro-invasive glaucoma surgeries (MIGS), has been developed. These procedures provide moderate IOP reduction and have a more favorable safety profile than filtering surgeries. MIGS procedures can be combined with phacoemulsification or with other non-filtering glaucoma procedures, such as endoscopic cyclophotocoagulation (ECP; BVI Endo Optiks, Waltham, MA, USA) [2].

The iStent *inject* trabecular micro-bypass (Glaukos Corp., San Clemente, CA, USA), often considered the most micro-invasive and safest of the MIGS implant devices, consists of two pre-loaded injectable titanium stents implanted via an ab-interno approach through the trabecular meshwork into Schlemm’s canal [14]. Once in position, the stents facilitate trabecular outflow. The prospective, randomized, multicenter, Phase 3 pivotal trial evaluating the device showed that iStent *inject* provides *a* ≥ 20% IOP reduction in 75.8% of OAG eyes at 24 months when used in combination with cataract surgery compared with 61.9% eyes undergoing cataract surgery alone [15]. For the treatment responders, 84% and 67% of the treated and control eyes, respectively, were medication-free at 23 months. Furthermore, an analysis of these iStent *inject* patients found improvements in quality of life in terms of ocular symptoms and vision-related activities compared with cataract surgery alone [16]. A large real-world cohort of Australian eyes, which included pseudoexfoliative (PXG) and pigmentary (PG) glaucoma, found clinically meaningful reductions in IOP and ocular hypotensive medication use at 1 year and 2 years [17, 18].

Endoscopic cyclophotocoagulation reduces aqueous production, thereby decreasing the inflow component of the inflow/outflow balance that contributes to IOP [19–21]. ECP is performed using an ab-interno approach wherein a power-titratable 810 nm laser probe is inserted through a limbal incision to allow visualization and continuous photocoagulation of the ciliary process epithelium over approximately 270° [19]. Our group previously reported that phacoemulsification with ECP was safe and effective as a primary procedure for combined cataract and glaucoma [22]. A prospective study by Francis et al. showed significant IOP reductions with cataract surgery plus ECP compared with cataract surgery alone [23]. ECP utility has been shown in mild-to-moderate glaucoma [19–21], as well as in refractory glaucoma [24].

Both ECP and iStent *inject* implantation have been shown to be effective at lowering IOP and topical ocular hypotensive medication burdens in OAG [15, 17, 18, 20, 21, 23]. ECP in combination with cataract surgery is a common treatment modality performed by surgeons in Brazil. With the increasing utilization of iStent *inject* trabecular micro-bypass, the combination of ECP and stent implantation has emerged as a potential treatment option, allowing the surgeon to target both outflow and inflow components of patients’ disease. Further, this study aims to address the paucity of clinical evidence in a Latin American population with glaucoma and cataract.

The present prospective multicenter study compares 1 year outcomes following phacoemulsification + ECP (Group 1) or phacoemulsification + ECP + iStent *inject* implantation (Group 2) in a Brazilian population The report supplies some of the first data on this treatment combination, with outcomes observed in a Latin American patient population that historically has been under-represented in the literature.

## Material and methods

### Study design

This was a prospective, comparative, multicenter case series that evaluated ECP with cataract surgery versus ECP plus iStent *inject* with cataract surgery in Brazilian patients with OAG. The study was performed in line with the principles of the Declaration of Helsinki [25]; all participants provided informed consent for their enrollment. The study was completed at two clinical sites: Centro Brasileiro De Cirurgia De Olhos (CBCO), Goiânia, GO, Brazil; and Centro Brasileiro Da Visão (CBY), Brasília, DF, Brazil. The study was reviewed and approved by the Ethics Committees of both hospitals. The study was registered in the Clinical Trial Database of the Federal University of Goiás, Brazil (CAAE ID# 20,053,019.5.0000.5078, Protocol #3.587.147, registered September 19, 2019).

Once enrolled, patients were prospectively randomized on a 1:1 basis to either phacoemulsification plus ECP or phacoemulsification plus ECP plus iStent *inject* implantation. An MS Excel (Microsoft, WA, USA) random number generator was used to provide the master randomization list. They were allowed to have one or both eyes treated as part of the study.

This study was pragmatic in nature, with the aim of gathering evidence for real-world treatment of glaucoma. Therefore, no preoperative or postoperative ocular hypotensive medication washout was required. Postoperative management of patients including medication reintroduction, management of adverse events, postoperative visit scheduling, and procedures were managed at the discretion of the treating physician using standard care.

### Inclusion criteria

Adults with OAG [primary open-angle glaucoma (POAG), pseudoexfoliation glaucoma (PXG), or pigmentary glaucoma (PG)] were included. The patients had to be candidates for ECP and iStent *inject* as judged by the investigators.

### Exclusion criteria

Eyes with prior glaucoma filtration surgery, angle-closure glaucoma, traumatic, malignant, uveitic, or neovascular glaucoma or other discernible congenital anomalies of the anterior chamber angle were not allowed to participate. Patients with retrobulbar tumor, thyroid eye disease, Sturge-Weber Syndrome or any other type of condition that may have caused elevated episcleral venous pressure were excluded. Study participation also was not allowed for any patient having a contraindicated ocular or systemic condition.

### Outcome measures

Outcome measures included IOP, topical ocular hypotensive medication burden, and safety. Data were collected and descriptive analyses were performed using MS Excel. The following measures were analyzed at postoperative study timepoints and compared to preoperative outcomes as appropriate: mean IOP; percentage of eyes with IOP ≤ 18 mmHg and with ≤ 15 mmHg; mean medication burden (number of medications) and percentage of eyes with categorical medication burden; percentage of eyes with more, fewer or the same medication burden at postoperative study timepoints compared to preoperative; Snellen best-corrected visual acuity; and percentage of eyes with intraoperative or postoperative complications. The schedule of visits and assessments through 12 months postoperative is shown in Supplemental Table S1.

## Statistics

Statistical calculations included mean, standard deviation, percentage of total, and categorical counts. Two-tailed *t*-tests were performed to compare postoperative results with preoperative results. A *p*-value of < 0.05 was considered significant.

### Sample size

Given the statistical assumptions (Supplemental Table S2) and a significance level of 0.05, 30 subjects per arm provided 80% power to detect a treatment difference that was statistically significant.

## Results

### Study participants

There were 35 eyes from 33 patients in the Phacoemulsification + ECP group (Group 1) and 36 eyes from 33 patients in the Phacoemulsification + ECP + iStent *inject* group (Group 2) (Table 1). Eyes in both treatment groups had generally comparable preoperative characteristics, including mean age, racial distribution, cup:disk ratio, visual fields, and retinal nerve fiber layer thickness. Nearly all eyes in both groups had moderate to severe primary open-angle glaucoma. More eyes in Group 2 had undergone laser iridotomy prior to the study. Both groups had mean preoperative IOP values of approximately 22 mmHg on 3.3–3.4 topical ocular hypotensive medications.

**Table 1:** Demographics and preoperative characteristics

Parameter	Phaco + ECP (group 1)	Phaco + ECP + iStent *inject* (group 2)	*p*-value (where applicable) group 1 versus group 2
*N* eyes (%)	35 (100)	36 (100)	
Mean age ± SD (years)	66.2 ± 9.3	68.7 ± 6.9	0.1738
Female/male (*n*)	18/17	21/15	
Race/ethnicity *n* (%)			
White	14 (40.0)	12 (33.3)	
Hispanic	11 (31.4)	12 (33.3)	
Black	8 (22.9)	10 (27.8)	
Asian	2 (5.7)	2 (5.6)	
Prior surgeries			
LTP	7 (20.0)	8 (22.2)	
LI	1 (2.9)	8 (22.2)	
Glaucoma type *n* (%)			
POAG	25 (71.4)	25 (69.4)	
PG	3 (8.6)	3 (8.3)	
NTG	1 (2.9)	0 (0)	
CMG	6 (17.1)	8 (22.2)	
Glaucoma severity			
Mild	2 (5.7)	2 (5.6)	
Moderate	18 (51.4)	15 (41.7)	
Severe	15 (42.9)	19 (52.8)	
Cup:disk ratio (mean ± SD)	0.8 ± 0.1	0.8 ± 0.1	0.4311
Visual field (MD) (mean ± SD)	− 9.49 ± 2.44	− 9.74 ± 2.75	0.4860
OCT RNFL (µm) (mean ± SD)	60 ± 10	62 ± 13	0.2304
Mean IOP (mmHg) ± SD	22.1 ± 3.6	22.0 ± 2.5	0.9317
Mean # medications ± SD	3.3 ± 0.6	3.4 ± 0.6	0.4408

*Phaco* Phacoemulsification; *CMG* Combined mechanism glaucoma; *ECP* Endoscopic cyclophotocoagulation; *IOP* Intraocular pressure; *LI* Laser iridotomy; *LTP* Laser trabeculoplasty; *MD* Mean defect; *NTG* Normal tension glaucoma; *PG* Pigmentary glaucoma; *POAG* Primary open-angle glaucoma; *RNFL* Retinal nerve fiber layer; *SD* Standard deviation

### Intraocular pressure

**Fig. 1:**
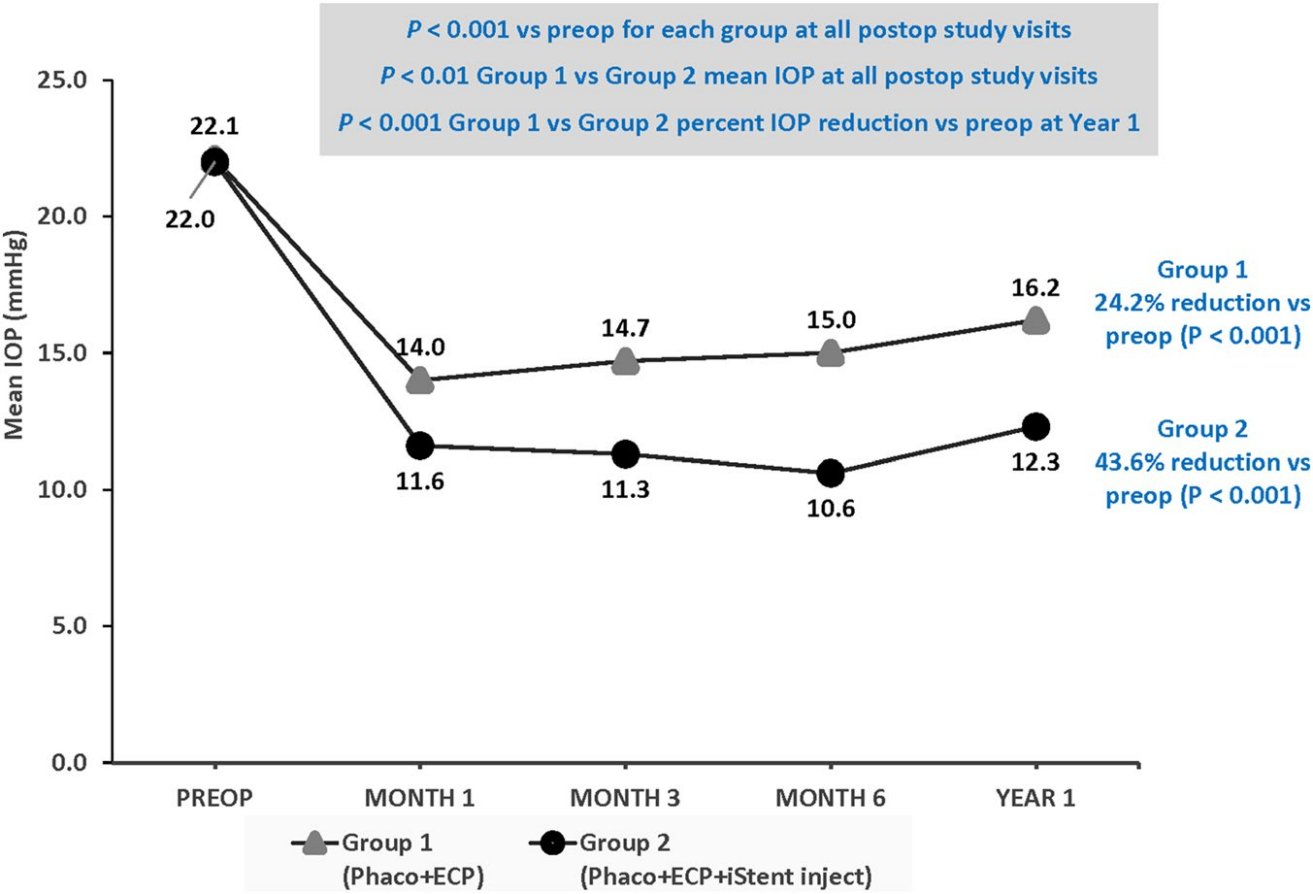
Intraocular pressure over the course of Year 1. *IOP* Intraocular pressure; *preop* Preoperative; *postop* Postoperative

Eyes in both groups achieved significant reductions in IOP at all postoperative data points versus preoperative (*p* < 0.001) (Fig. 1); IOP values were significantly lower in Group 2 versus Group 1 at all study visits through Year 1 (*p* < 0.01). At Year 1, eyes in Group 2 achieved significantly greater mean percent reductions in IOP from preoperative levels compared to Group 1 (43.6% versus 24.2%, respectively; *p* < 0.001) (Fig. 1).

Both treatment groups demonstrated clinically meaningful categorical reductions in IOP (Table 2). For Group 1, 11.4% and 0% of eyes had preoperative IOP ≤ 18 mmHg and ≤ 15 mmHg, respectively. At Year 1 these proportions had increased to 97.0% and 33.3%, respectively. Meanwhile, Group 2 had 2.8% and 0% of eyes with preoperative IOP ≤ 18 mmHg and ≤ 15 mmHg, respectively. Following surgery, 100% of Group 2 eyes achieved IOP ≤ 18 mmHg at all study visits through Year 1, and 100% achieved IOP ≤ 15 mmHg at visits from Month 3 to Year 1.

**Table 2:** Categorical reductions in IOP

	Preop	Month 1	Month 3	Month 6	Year 1
IOP ≤ 18 mmHg					
Group 1 *n* (%)	4 (11.4)	35 (100)	34 (100)	35 (100)	32 (97.0)
Group 1 total *n*	35	35	34	35	33
Group 2 *n* (%)	1 (2.8)	36 (100)	36 (100)	36 (100)	36 (100)
Group 2 total *n*	36	36	36	36	36
IOP ≤ 15 mmHg					
Group 1 *n* (%)	0 (0)	28 (80.0)	21 (61.8)	19 (54.3)	11 (33.3)
Group 2 *n* (%)	0 (0)	35 (97.2)	36 (100)	36 (100)	36 (100)

*ECP* Endoscopic cyclophotocoagulation; *IOP* Intraocular pressure

### Glaucoma medications

**Fig. 2:**
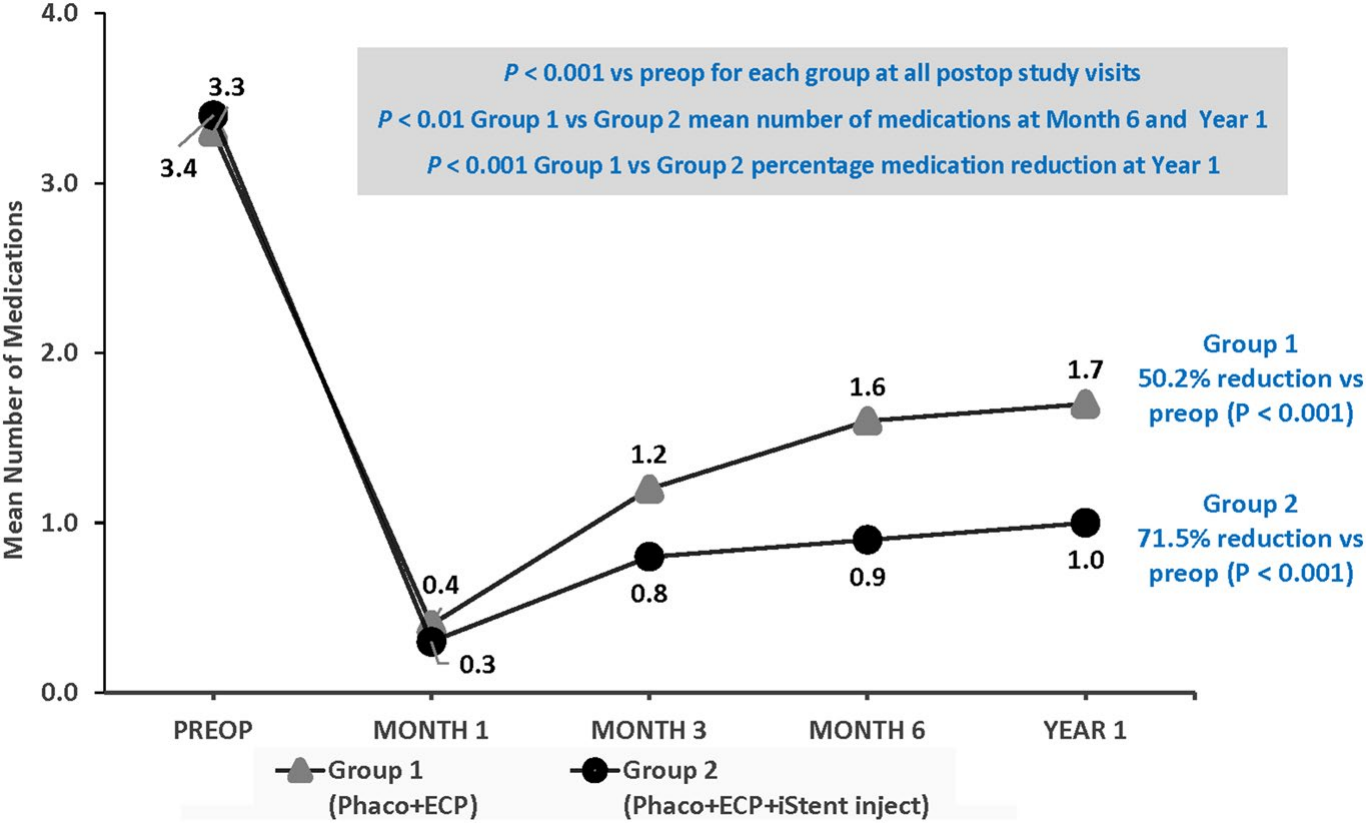
Topical ocular medication use over the course of Year 1. *IOP* Intraocular pressure; *preop* Preoperative; *postop* Postoperative

Both treatment groups achieved significant reductions in topical ocular medication use at all postoperative time points (*p* < 0.001) (Fig. 2). At Month 6 and Year 1, the mean numbers of medications were significantly lower in Group 2 than in Group 1 (*p* < 0.01). At Year 1, eyes in Group 2 achieved significantly greater medication reduction versus preoperative than those in Group 1 (71.5% versus 50.2%, respectively; *p* < 0.001) (Fig. 2).

**Fig. 3:**
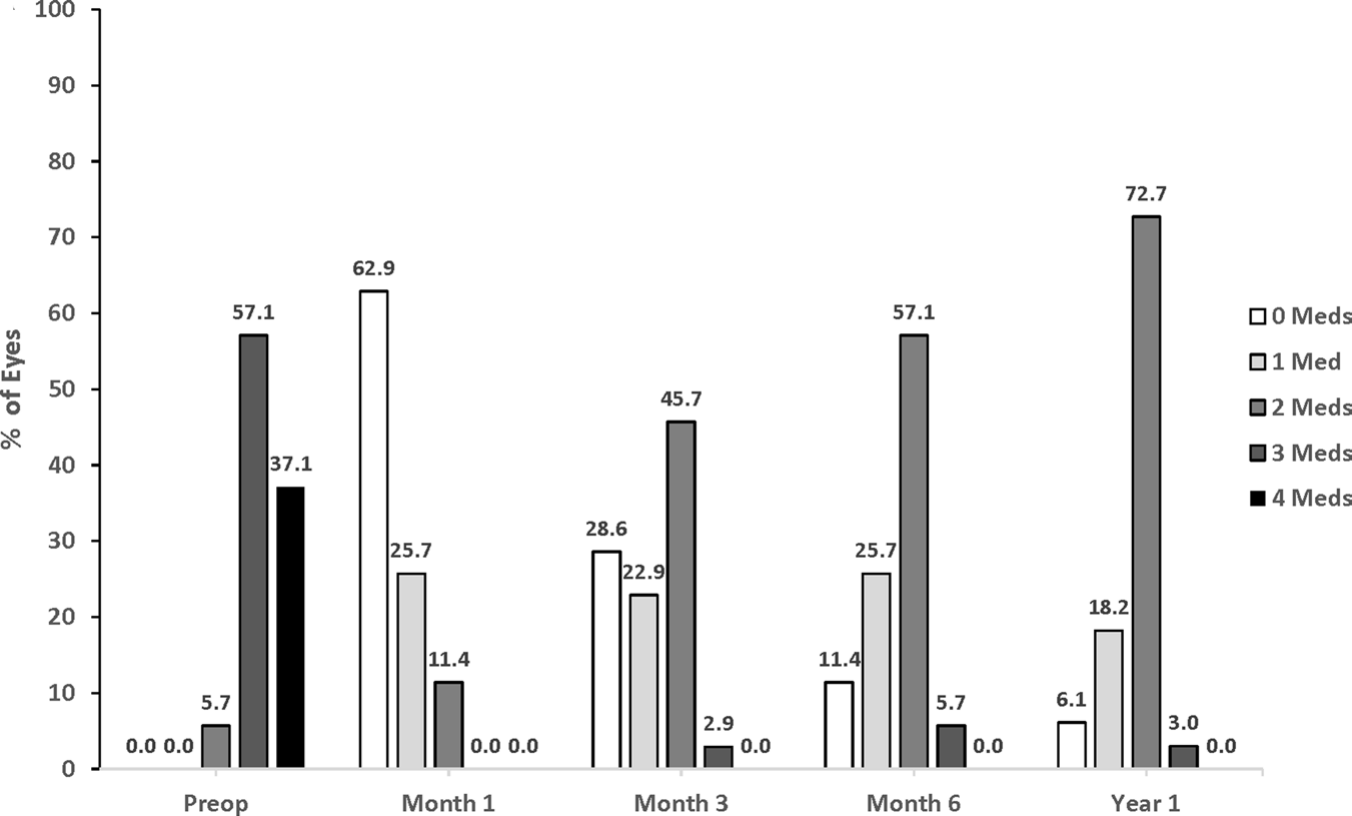
Proportion of eyes with categorical ocular hypotensive medication use in group 1 (Phacoemulsification + ECP). *ECP* Endoscopic cyclophotocoagulation; *meds* Medications; *preop* Preoperative

**Fig. 4:**
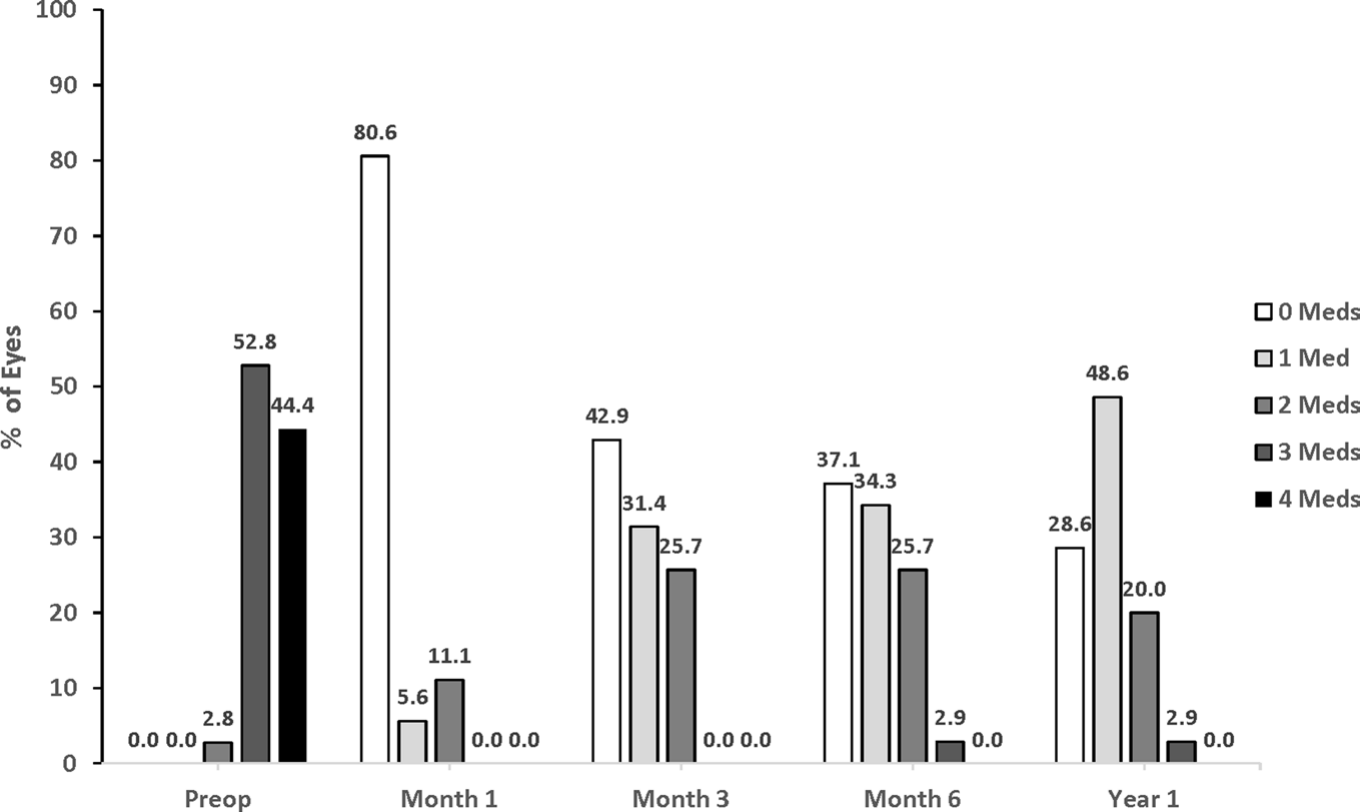
Proportion of eyes with categorical ocular hypotensive medication use in group 2 (Phacoemulsification + ECP + iStent *inject)*. *ECP* Endoscopic cyclophotocoagulation; *meds* Medications; *preop* Preoperative

At the preoperative visit in Group 1, no eyes were receiving 0 or 1 medication, while 94.2% of eyes were using 3–4 medications (Fig. 3). At Year 1, 6.1% and 18.2% of eyes were receiving 0 or 1 medication, respectively, while only 3.0% of eyes were on 3 medications; no eyes were receiving 4 medications. At the preoperative visit in Group 2, no eyes were receiving 0 or 1 medication, while 97.2% of eyes were using 3–4 medications (Fig. 4). At Year 1, 28.6% and 48.6% of eyes were receiving 0 or 1 medication, respectively, while only 2.9% of eyes were on 3 medications; no eyes were receiving 4 mediations.

**Fig. 5:**
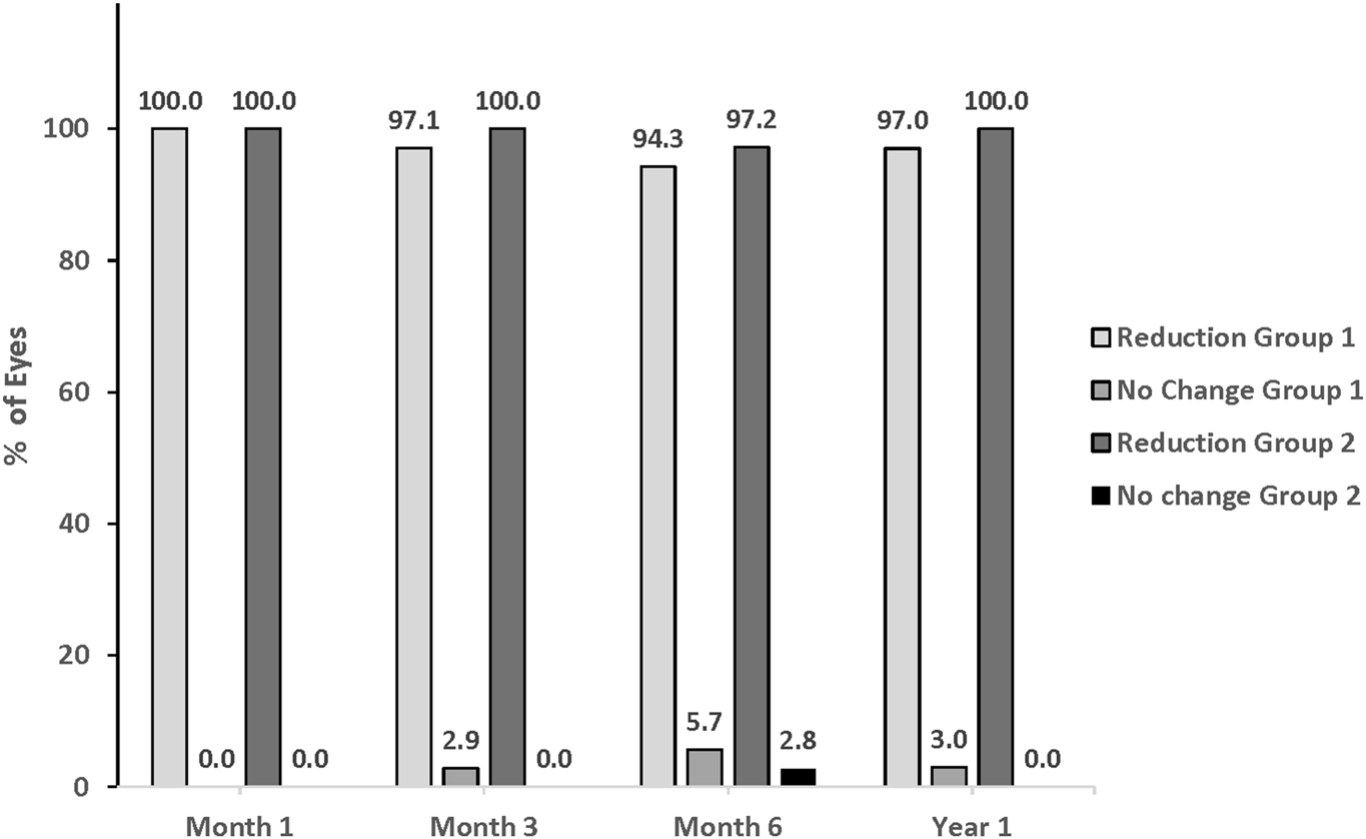
Change in categorical topical ocular medication use vs preoperative over the course of Year 1

Both treatment groups demonstrated high proportions of eyes with reductions in topical ocular hypotensive medication use throughout follow-up (Fig. 5). All eyes in both groups maintained or reduced their medication burden versus preoperative at all points in follow-up. None of the eyes in either group showed an increase in medication use.

### Safety

**Fig. 6:**
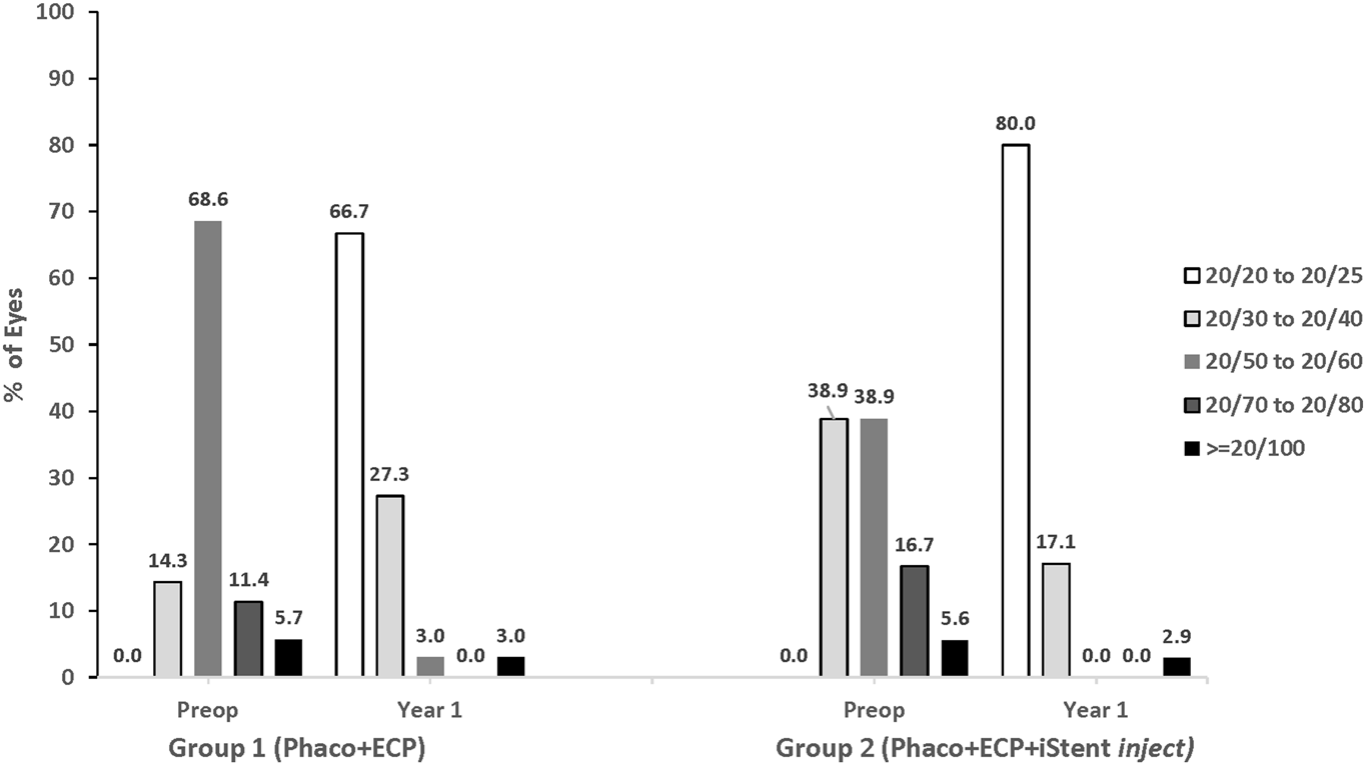
Categorical best-corrected visual acuity over the course of Year 1. *Phaco* Phacoemulsification; *ECP* Endoscopic cyclophotocoagulation

Best-corrected visual acuity was dramatically improved in both treatment groups (Fig. 6), consistent with expectations following standard cataract surgery. No eye in either group had BCVA of 20/20 or 20/25 preoperatively, but by Year 1, 68.6% and 80.0% of eyes had achieved BCVA of 20/20 to 20/25 in Group 1 and Group 2, respectively.

Intraoperative complications were few in both groups. These included one eye with transitory bleeding in each group and one eye in Group 2 with an over-implanted stent (Table 3). Postoperatively, there were modest numbers of eyes with mild cells and flare at Day 1 and Week 1 in both groups, consistent with levels expected after standard cataract surgery. Adverse events were generally minimal, few, and resolved without sequelae.

**Table 3:** Safety parameters at all study visits

*N* (%)	Intraoperative	Day 1	Week 1	Month 1	Month 3	Month 6	Year 1
*Group* 1							
None	34 (97.1)	28 (80.0)	31 (88.6)	33 (94.3)	34 (97.1)	34 (97.1)	32 (97.0)
Transitory bleeding	1 (2.9)	N/A	N/A	N/A	N/A	N/A	N/A
Cells and flare							
1/4+	N/A	2 (5.7)	4 (11.4)	1 (2.9)	0 (0)	0 (0)	0 (0)
2/4+	N/A	3 (8.6)	0 (0)	0 (0)	0 (0)	0 (0)	0 (0)
IOP spike, no sequelae	N/A	1 (2.9)	0 (0)	0 (0)	0 (0)	0 (0)	0 (0)
Lost fixation	N/A	0 (0)	0 (0)	1 (2.9)	1 (2.9)	1 (2.9)	1 (3.0)
*Group* 2							
None	34 (94.4)	23 (63.9)	29 (80.6)	35 (97.2)	35 (97.2)	35 (97.2)	34 (97.1)
Transitory bleeding	1 (2.8)	N/A	N/A	N/A	N/A	N/A	N/A
Stent over-implanted	1 (2.8)	N/A	N/A	N/A	N/A	N/A	N/A
Cells and flare							
1/4+	N/A	10 (27.8)	7 (19.4)	0 (0)	0 (0)	0 (0)	0 (0)
2/4+	N/A	2 (5.6)	0 (0)	0 (0)	0 (0)	0 (0)	0 (0)
IOP spike, no sequelae	N/A	0 (0)	0 (0)	0 (0)	0 (0)	0 (0)	0 (0)
Hyphema 2+ *		1 (2.8)	0 (0)	0 (0)	0 (0)	0 (0)	0 (0)
Lost fixation	N/A	0 (0)	0 (0)	1 (2.8)	1 (2.8)	1 (2.8)	1 (2.9)

*no sequelae, no intervention needed

*ECP* Endoscopic cyclophotocoagulation; *IOP* Intraocular pressure

Cup:disk ratios, visual fields, and retinal nerve fiber layer and corneal thicknesses remained stable over the course of the 1-year study in both groups (Table 4).

**Table 4:** Additional safety parameters at the preoperative and Year 1 visits

Parameter	Group 1 Preop	Group 1 Year 1	Group 2 Preop	Group 2 Year 1
*N* eyes	35	33	36	35
Cup:disk ratio ± SD	0.8 ± 0.1	0.8 ± 0.1	0.8 ± 0.1	0.8 ± 0.1
Visual field (MD) ± SD	− 9.49 ± 2.44	− 8.75 ± 3.11	− 9.74 ± 2.75	− 9.00 ± 2.87
OCT RNFL (µm) ± SD	60 ± 10	58 ± 11	62 ± 13	60 ± 14

*ECP* Endoscopic cyclophotocoagulation; *MD* Mean defect; *OCT* Optical coherence tomography; *RNFL* Retinal nerve fiber layer; *SD* Standard deviation

## Discussion

A considerable shift from traditional glaucoma surgeries to MIGS procedures has occurred in recent years [26, 27]. A study from the American Academy of Ophthalmology Intelligent Research in Sight (IRIS®) Registry data found that, from 2013 to 2018, MIGS surgeries rose to comprise nearly half of all glaucoma surgeries in the US. The most common concurrent procedures were ECP and iStent/iStent *inject* implantation, which accounted for 55.4% of all concurrent glaucoma procedures [26]. These trends underscore the relevance and value of the current study.

The present report provides safety and effectiveness outcomes of phacoemulsification and ECP, either with or without iStent *inject* implantation, in Brazilian patients with cataract and glaucoma. Substantial IOP-lowering and reductions in ocular hypotensive medication use were observed for both treatment groups and were sustained through 1 year. Both procedures were generally well-tolerated with few procedure-related adverse events.

The combination of micro-bypass stent, cataract surgery, and ECP leverages different mechanisms of action in glaucoma management [28]. Not unexpectedly, the eyes receiving the iStent *inject* in combination with phacoemulsification and ECP demonstrated significantly greater IOP reductions throughout the 1 year of follow-up (*p* < 0.01). Similarly, although both treatment groups achieved clinically significant categorical reductions in IOP versus preoperative, reductions were more marked in the group undergoing iStent *inject* implantation (Group 2), including a three-fold higher percentage of eyes achieving IOP ≤ 15 mmHg in Group 2 versus Group 1 at Year 1. Additionally, the IOP reductions in Group 2 appeared to be maintained with less upward drift over time than for those undergoing the single ECP procedure.

These findings are consistent with the literature on ECP and trabecular micro-bypass. A retrospective consecutive case series by Ferguson et al. [28] demonstrated significantly greater IOP reductions in eyes receiving ECP + iStent than those undergoing iStent implantation alone. Similarly, a longitudinal retrospective 12 month study by Pantalon and colleagues [29] in eyes with early-to-moderate OAG reported significantly larger IOP reductions from baseline for eyes undergoing phacoemulsification + ECP + 2 iStents versus phacoemulsification + 2 iStents. These results demonstrate the value of pairing procedures and are consistent with the current study in which the combined procedure provided greater IOP-lowering efficacy than cataract surgery with ECP alone.

Favorable trends also were observed in medication reduction in the current study, with the vast majority of eyes in both groups reducing their number of medications through Year 1. As with IOP, the medication reductions were significantly greater in the group undergoing iStent *inject* implantation (Group 2). This included a three-fold higher proportion of Group 2 versus Group 1 eyes on 0–1 medication at Year 1, and a threefold to fourfold lower proportion of Group 2 eyes on ≥ 2 medications at Year 1. These outcomes are aligned with those of Pantalon and colleagues, who reported significantly greater 1-year medication reductions after phacoemulsification + ECP + iStent *inject* than after phacoemulsification + iStent *inject* [29].

These medication reductions represent significant benefits at the level of each patient, given the well-known personal, physical, social, and financial consequences of chronic medication exposure. For example, the cost of chronic medication can be a financial burden for patients; not surprisingly, numerous studies have shown the cost effectiveness of the iStent device compared with the use of chronic topical medications [30–36]. Topical ocular hypotensive medications also can contain preservatives, such as benzalkonium chloride, which are known to cause adverse effects on the ocular surface [4, 11]. In addition, patient adherence to topical ocular hypotensive drops is typically less than ideal [7, 9, 10, 37, 38] for a number of potential reasons (e.g., complex medication regimens, difficulty with drop instillation, or social and environmental limitations) [9]. Poor adherence to glaucoma medication therapy can put a patient at risk for glaucoma progression with irreversible optic nerve damage and vision loss. Thus the implantation of micro-bypass stents can provide a treatment alternative that reduces preservative load to the ocular surface [18, 39–45] and lessens reliance on patient adherence, thereby helping to preserve visual function.

Both ECP and iStent *inject* implantation were associated with few intraoperative complications or postoperative adverse events in our study. The majority of events occurred in the initial days following surgery and resolved soon thereafter. Cup:disk ratios, visual fields, and retinal nerve fiber layer thickness remained stable over the course of the 1 year study in both groups. Best-corrected visual acuity improved dramatically at Year 1 versus preoperative, consistent with what would be expected after cataract surgery alone; there was no indication that the addition of iStent *inject* or ECP detracted from patients’ overall visual potential.

These visual acuity findings are consistent with prior evaluations of phacoemulsification + ECP and/or iStent implantation [22, 29, 46–51]. For example, a previous study from our group demonstrated improvements in the logMAR visual acuity (*p* = 0.01) for up to 2 years in eyes receiving phacoemulsification + ECP [22]. Morales and colleagues [50] found improvements in corrected distance visual acuity of 2 Snellen lines or more at 1 year in 73% of patients undergoing ECP and phacoemulsification. In another study of eyes undergoing ECP with phacoemulsification, Clement et al. [47] reported 94% of eyes achieved stable or improved vision after 1 year. Kang et al. [48] demonstrated maintenance or improvement in visual acuity for 95% of their ECP + phacoemulsification group with a mean follow-up of 21 months. Several studies have shown improvements in visual acuity 1 year or more following iStent implantation, either as a stand-alone procedure [51], or when combined with cataract surgery [46, 49]. Finally, Pantalon et al. [29] reported similar 1 year BCVA results in eyes undergoing phacoemulsification plus either iStent *inject* + ECP or iStent *inject*. All of these studies, as well as the current one, demonstrate favorable outcomes with ECP, iStent *inject* implantation, or the combination of the two procedures in combination with phacoemulsification in terms of preserving the visual improvements expected after cataract surgery alone.

This study is limited by its modest sample size, 1 year follow-up duration, and inclusion of data from 2 surgeons at 2 sites. Patients with other forms of glaucoma than OAG were excluded, as well as those who had undergone previous glaucoma filtration surgery. The majority of patients in the study population were White, Hispanic, or Black, so results may not be directly applicable to other demographic groups (e.g., Asians). Not all data were available for all parameters at all time points. A future study could include a greater number of patients, longer duration of follow-up, data from more sites, or a broader range of glaucoma subtypes.

## Conclusion

When combined with cataract surgery, both ECP and ECP + iStent *inject* implantation procedures were safe and effective in lowering IOP, reducing topical ocular hypotensive medication use, and preserving the visual improvements experienced after cataract surgery. The IOP and medication reductions were greater in the group undergoing iStent *inject* implantation alongside phacoemulsification + ECP than in the group receiving phacoemulsification + ECP only. Thus, the results demonstrate favorable outcomes with these two procedures when combined with cataract surgery, as well as confirmation of the additional benefit of stent implantation, through one year postoperative in a Brazilian patient cohort.

## Supplementary Information

Below is the link to the electronic supplementary material.

Supplementary file1 (DOCX 28 kb)

## Data Availability

All authors had full access to all of the data in this study and take complete responsibility for the integrity of the data and accuracy of the data analysis.
