# Supplementary material for: A multicenter prospective comparative study evaluating cataract surgery and endoscopic cyclophotocoagulation either with or without iStent inject implantation in Brazilian patients with glaucoma
Source: Int Ophthalmol. 2022 Oct 23;43(5):1665–76. doi: 10.1007/s10792-022-02563-4 (PMC10149466; doi:10.1007/s10792-022-02563-4)
Supplement: Supplementary file 1 — Supplementary file1 (DOCX 28 kb) [file 10792_2022_2563_MOESM1_ESM.docx]

**Supplemental Table 1. Schedule of Visits and Assessments**

|  | **Preop** | **Operative** | **Day 1** | **Week 1** | **Month 1** | **Month 3** | **Month 6** | **Month 12** |
| --- | --- | --- | --- | --- | --- | --- | --- | --- |
| **Informed consent, demographics, ophthalmic/medical history** | X |  |  |  |  |  |  |  |
| **Glaucoma medications** | X |  | X | X | X | X | X | X |
| **BCVA (pinhole if needed)** | X |  | X | X | X | X | X | X |
| **IOP (GAT)** | X |  | X | X | X | X | X | X |
| **Slit lamp exam*** | X |  | X | X | X | X | X | X |
| **Pachymetry** | X |  |  |  |  |  |  | X |
| **C/D ratio*** | X |  |  |  |  |  |  | X |
| **Optic nerve assessment*** | X |  |  |  |  |  |  | X |
| **Visual field*** | X |  |  |  |  |  | X | X |
| **Gonioscopy*** | X |  |  |  |  |  |  | X |
| **Surgical complications and Adverse events** |  | X | X | X | X | X | X | X |
| **Randomization** |  | X |  |  |  |  |  |  |
| **Phacoemulsification with ECP +/- iStent *inject*** |  | X |  |  |  |  |  |  |

*For visits not marked by “X,” the frequency of measurement was at the discretion of the surgeon.

BCVA = best-corrected visual acuity; C/D = cup:disc ratio; ECP = endoscopic cyclophotocoagulation; GAT = Goldmann applanation tonometry; IOP = intraocular pressure; Preop = preoperative.

**Supplemental Table 2. Assumptions for Statistical Analysis**

| Primary Endpoint | - At 12 months |
| --- | --- |
| At 12 months | - After ECP + phacoemsulfication (Group 1), the mean IOP reduction from preoperative would be ~2 mmHg - After ECP + iStent *inject* + phacoemulsification (Group 2), the mean IOP reduction from preoperative would be ≥5 mmHg |
| Standard deviation (SD) | - SD for the change from preoperative in both treatment arms would be ≤4 mmHg |

Note: the analysis was based on Month 12 change from preoperative IOP, not IOP at month 12. There is no estimate of the standard deviation available. The ECP+phacoemulsification data assumptions are based on the publication by Francis et al.^23^ which demonstrated mean IOP reductions from preoperative at 6 months = 2.5 mmHg (from 18.1 to 15.6) and at 12 months = ~2 mmHg (from 18.1 to 16.0).

The ECP+iStent *inject*+phacoemulsification assumptions were based on a study by Pantalon et al,^29^ which showed a mean IOP reduction of 6.93 mmHg at Month 12 versus preoperative (from 19.97 to 13.05 mmHg); and a study by Ferguson et al,^28^ which showed a mean IOP reduction of 7.14 mmHg at Month 12 versus preoperative (from 21.49 to 14.35 mmHg).
